# Clinicopathological and genetic study of a rare occurrence: Malignant transformation of fibrous dysplasia of the jaws

**DOI:** 10.1002/mgg3.1861

**Published:** 2022-01-05

**Authors:** Ruirui Shi, Xuefen Li, Jianyun Zhang, Feng Chen, Ming Ma, Yanrui Feng, Tiejun Li

**Affiliations:** ^1^ Central Laboratory Peking University School and Hospital of Stomatology & National Center of Stomatology & National Clinical Research Center for Oral Diseases & National Engineering Laboratory for Digital and Material Technology of Stomatology & Beijing Key Laboratory of Digital Stomatology & Research Center of Engineering and Technology for Computerized Dentistry Ministry of Health & NMPA Key Laboratory for Dental Materials Beijing PR China; ^2^ Research Unit of Precision Pathologic Diagnosis in Tumors of the Oral and Maxillofacial Regions Chinese Academy of Medical Sciences (2019RU034) Beijing China; ^3^ Department of Oral Pathology Peking University School and Hospital of Stomatology & National Center of Stomatology & National Clinical Research Center for Oral Diseases & National Engineering Laboratory for Digital and Material Technology of Stomatology & Beijing Key Laboratory of Digital Stomatology & Research Center of Engineering and Technology for Computerized Dentistry Ministry of Health & NMPA Key Laboratory for Dental Materials Beijing PR China

**Keywords:** copy number alterations, fibrous dysplasia, *GNAS*, malignant transformation, *TP53*

## Abstract

**Background:**

Malignant transformation of fibrous dysplasia (FD) is very rare and little is known about this occurrence.

**Methods:**

We present the detailed clinical course of three cases of osteosarcoma arising from FD of the jaws and explore the genetic aberrations by Sanger sequencing, whole‐exome sequencing (WES) and immunohistochemistry (IHC). A literature review of important topics related to this occurrence was also performed.

**Results:**

It was observed that patients with secondary sarcoma from FD showed a wide range of ages, with most during the third decade. Female and males were equally affected. Craniofacial bones and femurs were the most affected sites. High‐risk factors for this occurrence included polyostotic FD, McCune‐Albright syndrome and excess growth hormone. Notably, a potential relationship between thyroid hormones and sarcoma development was suggested in one patient, who began to show malignant features after hypothyroidism correction. Sanger sequencing revealed *GNAS* mutations of FD retained in all malignant tissues. Additionally, abnormal *TP53* was demonstrated in all three cases by WES and IHC. WES also revealed two other driver mutations, *ROS1* and *CHD8*, and large amounts of somatic copy number alterations (CNAs) where various oncogenes and tumour suppressors are located.

**Conclusion:**

This study demonstrated and reviewed the clinical features and risk factors for a rare occurrence, secondary sarcoma from FD, and provided important new knowledge about its genetics.

## Graphical abstract

Whole exome sequencing revealed the underlying genetic mechanisms of osteosarcoma arising from fibrous dysplasia of the jaws. Significant somatic single nucleotide variations, including TP53, ROS1 AND CHD8, as well as large amounts of somatic copy number alterations were demonstrated.

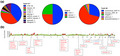

## 1 INTRODUCTION

Fibrous dysplasia (FD) is a skeletal disorder arising from somatic mutations in *GNAS* (OMIM *139320) and is associated with bone marrow stromal cells (BMSCs; Saggio, 2019). *GNAS* mutations in FD are gain‐of‐function alterations, which constitutively activate adenylyl cyclase (AC) to generate excess cyclic adenosine monophosphate (cAMP) through loss of GTPase activity of GTP‐bound Gαs (Landis et al., 1989) and abnormal activation of GDP‐bound Gαs (Hu & Shokat, 2018), resulting in abnormal proliferation and differentiation of mutation‐bearing BMSCs (Marie, 2001).

In FD, normal bone and bone marrow are replaced by abnormal trabeculae and fibrous tissue, with enhanced osteoclastogenesis, devoid of haematopoiesis and adipogenesis (Lichtenstein, 1938; Riminucci et al., 1999, 2003). The fibrotic area of FD consists of cells with phenotypic features of pre‐osteogenic cells, whereas the lesional bone formed de novo within fibrous areas represents the biosynthetic output of mature but abnormal osteoblasts (Riminucci et al., 1997). The bone trabeculae within the fibrous tissues are heterogeneous in overall amount, cellularity, structure and architecture (Riminucci et al., 1997, 1999).

The clinical presentation of FD demonstrates an extensive spectrum. Based on the amount of affected bone and whether it is accompanied by extraskeletal manifestations, FD is generally categorized into three forms: monostotic or polyostotic FD and McCune‐Albright syndrome (MAS, polyostotic FD with café‐au‐lait macules and/or hyperfunctioning endocrinopathies; Boyce & Collins, 2020; Lichtenstein, 1942).

FD itself is a benign disease, however, with a variable clinical course (Han et al., 2014; Sweeney & Kaban, 2020). Malignant transformation is one of the most damaging courses. Ruggieri and coworkers reported a mortality rate of 53.6% in FD patients with malignant transformation (Ruggieri et al., 1994). According to a recent report focused on the prognosis of malignant transformation of FD in craniofacial bones, it was confirmed that the prognosis was disappointing, with a median survival ranging from 4 to 62 months (Li et al., 2020). All forms of FD, namely, monostotic and polyostotic FD and MAS, can transform into sarcomas (Li et al., 2020; Ruggieri et al., 1994). The malignant transformations reported in the literature include osteosarcoma, fibrosarcoma, chondrosarcoma, malignant fibrous cell tumours, and angiosarcoma (Fukuroku et al., 1999; Li et al., 2020; Ruggieri et al., 1994). Among them, osteosarcoma is the most frequently occurring histologic type, with a frequency of 48%–70% in the literature (Li et al., 2020; Riddle & Bui, 2013; Ruggieri et al., 1994; Schwartz & Alpert, 1964; Yabut et al., 1988). Malignancy is suggested by rapid growth, pain, or a significant rapid change in radiographic appearance, especially in mineralization (Qu et al., 2015; Ruggieri et al., 1994). Computed tomography scans can be helpful in recognizing malignancy as well as in determining their extent (Riddle & Bui, 2013). Microscopically, the features of FD‐derived sarcoma showed no difference from those in normal bone (Schwartz & Alpert, 1964).

It has been reported that the malignancy of FD ranges from 0.4% to 4% (Schwartz & Alpert, 1964). Due to its rarity, its clinical characteristics and molecular pathogenesis remain largely unclear. To obtain a better understanding of this rare but damaging disorder, we reported three new cases and performed whole‐exome sequencing (WES) using both benign FD tissues and malignant tissues in the present study. A literature review regarding the limited known knowledge about the genetic aberrations associated with sarcoma change of FD, a rare entity, and several important topics related to its clinical features was also conducted.

## 2 MATERIALS AND METHODS

### 2.1 Patients and specimens

Three patients at Peking University School and Hospital of Stomatology with malignant tumours arising from FD were enrolled. Their clinical information, including age, sex, symptoms, serum alkaline phosphatase (ALP) level, clinical history and radiographic examinations, were retrieved from their medical records and reviewed retrospectively in detail. Haematoxylin–eosin (H&E) stained sections of formalin‐fixed paraffin‐embedded tissue were examined for pathological evaluation by three specialized pathologists.

### 2.2 DNA extraction

Genomic DNA was extracted from both the FD tissues and the matched malignant tissues of each patient using QIAamp DNA Mini Kit (Qiagen) according to the manufacturer's instructions. For patient #1, formalin‐fixed tissue prior to decalcification was used, whereas formalin‐fixed paraffin‐embedded (FFPE) sections from decalcified tissues were used for the other two patients. NanoDrop8000 (ThermoFisher Scientific) and Qubit2.0 fluorometer (ThermoFisher Scientific) were used to assess the quality and quantity of DNA.

### 2.3 *GNAS* mutation analysis

Sanger sequencing of regular PCR‐amplified exon 8 and exon 9 of the *GNAS* (GenBank and NCBI reference sequence: AH002748.2/NG_016194.2/NM_000516.7) gene was performed as described previously (Kuznetsov et al., 2008).

### 2.4 Whole‐exome sequencing

Qualified DNA for WES was obtained from patient #1. For WES, DNA was sheared into 180–200 bp segments and submitted to library preparation with an Agilent SureSelect Human All Exon V5/V6 kit (Agilent Technology) according to the manufacturer's protocol. Paired‐end sequencing (150 bp) was conducted on an Illumina HiSeq platform (Novogene). At least 14 giga bases of raw data were produced for each sample.

After quality control of raw data, clean sequencing reads were mapped to the human reference genome (human_B37) using BWA (Li & Durbin, 2009) and Samblaster (Faust & Hall, 2014). Through comparison with matched FD tissue data, somatic single nucleotide variations (SNVs) and insertions/deletions (INDELs) specific to malignant specimens were identified from malignant tumours using MuTect (Cibulskis et al., 2013) and Strelka (Saunders et al., 2012), respectively. Functional annotation of somatic mutations on their encoded amino acids was performed using ANNOVAR. By comparison with known driver genes using in‐house software, driver genes of malignancy were identified. Somatic copy number alterations (CNAs) were identified using control‐FREEC software (Boeva et al., 2012).

### 2.5 Immunohistochemistry

For immunohistochemistry (IHC), FFPE tissues of both benign FD and sarcoma specimens were sectioned at a thickness of 4 µm. They were deparaffinized using xylene and hydrated through graded alcohols. For antigen unmasking, sections were treated with citrate buffer (pH 6.0). Sections were incubated with mouse monoclonal p53 antibody (ZM‐0408, ZSGB‐BIO) at 4°C overnight, followed by treatment with PV9001 (ZM‐0408, ZSGB‐BIO) and visualization with DAB (ZM‐0408, ZSGB‐BIO). Negative controls were obtained by omitting the primary antibody.

## 3 RESULTS

### 3.1 The clinicopathological data of three patients with malignant transformation of FD

Three patients with osteosarcoma arising from FD of the jaws were identified from a total of 253 patients with FD in the maxillofacial bones over a duration of 20 years (2000–2020). The clinicopathological information of these patients was summarized in Table 1.

**TABLE 1:** The clinicopathological information of patients in this study

No. of patients	Gender	Age (years)	Duration	Type of FD	Site of malignant transformation	Serum ALP (U/L) (value of the healthy)	Pathology of malignant tissue
1	F	39	≥30 years	PFD (craniofacial bones and rib)	Mandible	247 (35–100)	OS
2	M	56	≥10 months	MFD (condyle)	Condyle	265 (30–110)	OS
3	F	31	≥18 years	MAS (PFD, precocious puberty, skin pigmentation)	Mandible	1323 (30–110)	OS

Abbreviations: F, female; M, male; MFD, monostotic fibrous dysplasia; MAS, MuCune‐Albright syndrome; OS, osteosarcoma; PFD, polyostotic fibrous dysplasia.

**FIGURE 1:**
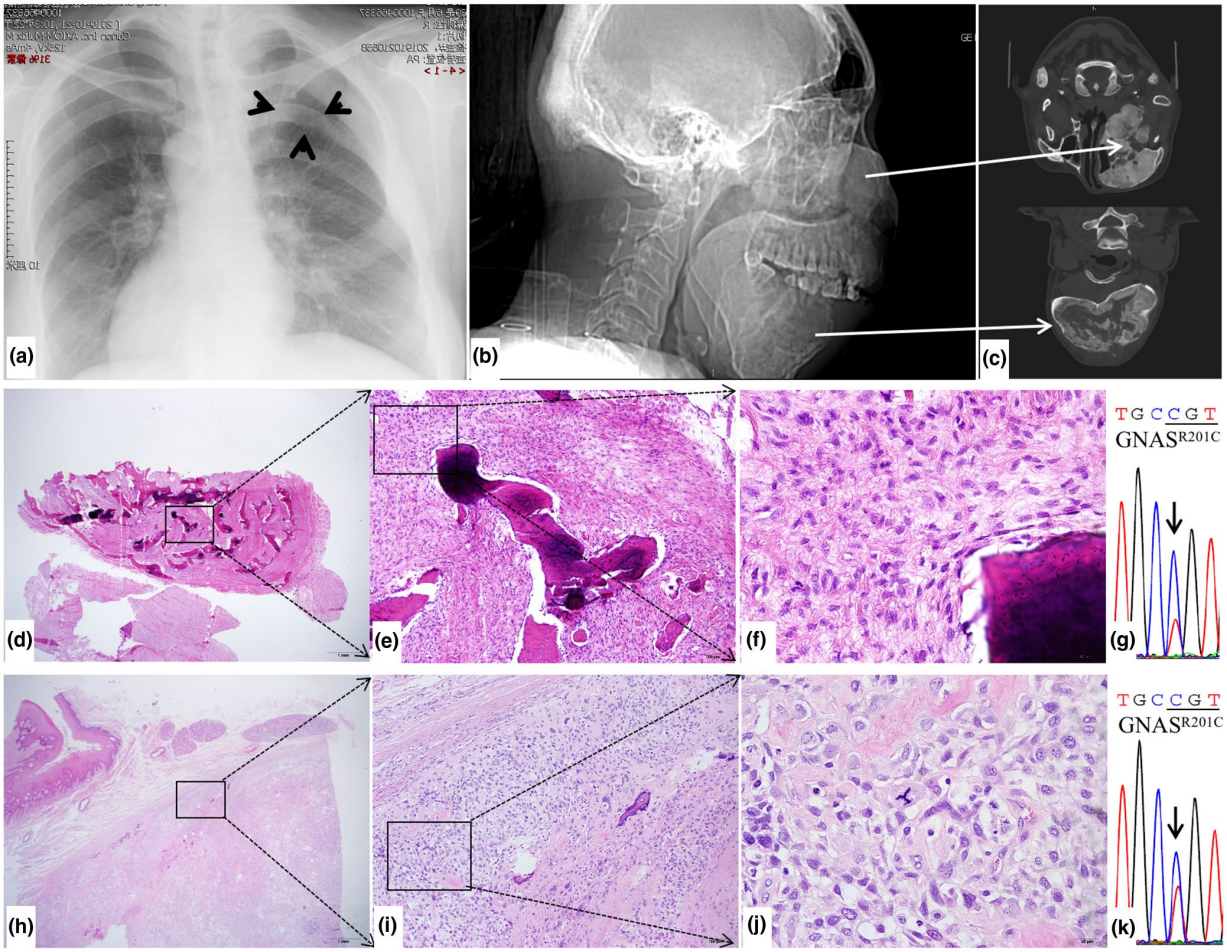
Radiologic‐pathological images and *GNAS* mutation analysis of patient #1. (a) X‐ray chest film showed radiopaque widening in the right fourth rib and disappearance of the medulla, indicated by arrow heads. (b) Right lateral view of head CT showed abnormal swelling of multiple craniofacial bones. (c) CT cross sections demonstrated an ill‐defined mass with a ground‐glass appearance involving maxilla as well as multiple adjacent bones (top) and mandible (bottom). Thinning of cortical bone and narrowing of the left maxillary sinus and nasal cavity can also be observed. (d–f) Histopathologic images of the benign tissue showed FD features: fibrous tissue and immature trabeculae (d: 4X view, e: 10X view, f: 40X view). (g) *GNAS* mutation analysis of the benign FD tissue from patient #1 revealed an R201C mutation, with a change from C in site 601 of cDNA to T. (h–j) Histopathological images of the osteosarcoma showed obvious hyperchromatic and pleomorphic nuclei, with bone‐like tissue observed (h: 4X view, i: 10X view, j: 40X view). (k) *GNAS* mutation analysis of malignant tissues also presented the R201C mutation

The first patient was a 39‐year‐old female patient, who presented with complaints of facial swelling for more than 30 years. She underwent three operations at the ages of 9, 19 and 25 years old in external hospitals due to a gradual increase in swelling and was consistently diagnosed with FD. Three months before her referral to our hospital, she was found to have hypothyroidism and Euthyrox was prescribed. It was noted that since being treated with medicine for hypothyroidism, her facial swelling began to grow rapidly, with significant tooth displacement and proptosis of her left eye. Routine blood tests revealed an increased serum ALP level of 247 U/L. A CT scan showed an ill‐defined mass with a ground‐glass appearance involving her left zygomatic, ethmoid, sphenoid, temple and maxillary bones and mandible extending from the right body to the left condyle. Thinning of cortical bone and narrowing of the left maxillary sinus and nasal cavity were also observed (Figure 1b,c). X‐ray chest film showed radiopaque widening in the right fourth rib and disappearance of the medullary cavity (Figure 1a). There was no significant disorder suggestive of MAS. Contour correction of the left maxilla and segmental osteotomy of the mandible were carried out, and histopathological analysis was performed, which showed FD (Figure 1d–f) in the maxilla and an osteosarcoma lesion in the mandible (Figure 1h–j).

**FIGURE 2:**
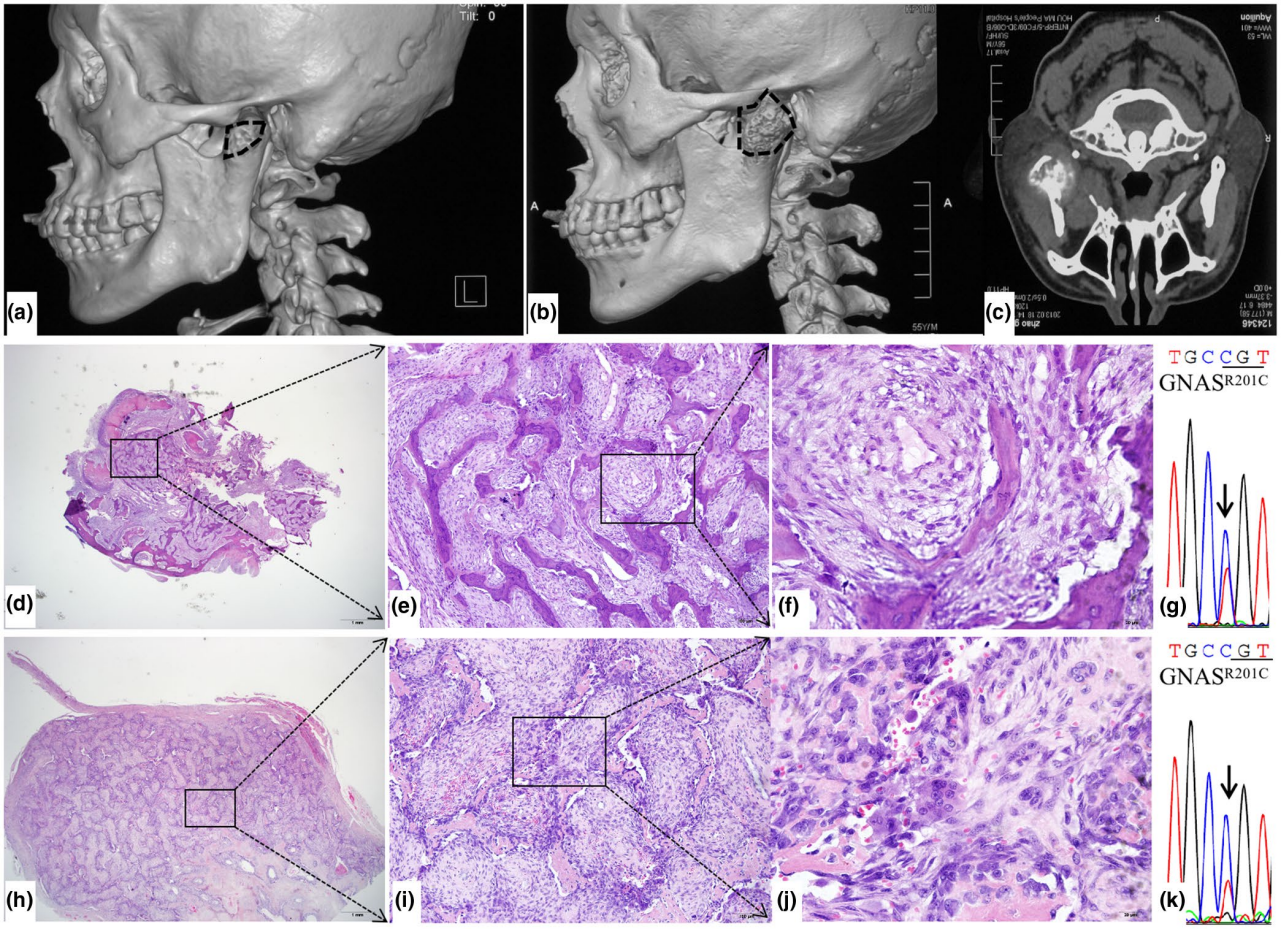
Radiologic‐pathological images and *GNAS* mutation analysis of patient #2. (a) Left lateral view of head CT performed on his first reference. The lesion of the condyle is highlighted by a dotted circle. (b) The condylar lesion became larger 3 months after the first operation. (c) CT cross section shows the mixed radiopaque and radiolucent mass in the left condyle. (d–f) Representative histological images of the benign tissue composed of cellular active fibrous stroma with woven trabeculae (d: 4X view, e: 10X view, f: 40X view). (g) *GNAS* mutation analysis of benign FD tissues from patient #2 demonstrated R201C mutation. (h–j) Histological images of sarcomatous stroma, trabeculae of bone and active multinucleated giant cells, representing an osteosarcoma diagnosis (h: 4X view, i: 10X view, j: 40X view). (k) A same R201C mutation was also revealed for the malignant tissues of this patient

Patient #2 was a 56‐year‐old male, who was referred to our hospital due to progressively worsening pain in the front area of the left ear (Figure 2a), with obvious swelling and limitation of mouth opening for 10 months. With the uncertainty of the nature of the disease, an incisional biopsy was carried out, and histopathological analysis revealed a diagnosis of FD, with a very low risk of malignant transformation, composed of bone and fibrous tissue (Figure 2d–f). The mass was found to be significantly increased three months postoperatively, as demonstrated on CT examination, measuring 5 cm in the buccal lingual direction and 4.6 cm in the axial direction, with numbness (Figure 2b,c). Blood tests revealed an elevated level of ALP of 265 U/L. Segmental osteotomy of the left mandible involving the lesion of the condyle was then conducted, and pathological analysis revealed a more proliferating area with many osteoclasts and atypical nuclei, which is the manifestation of osteosarcoma of the osteoclast‐rich type (Figure 2h–j).

**FIGURE 3:**
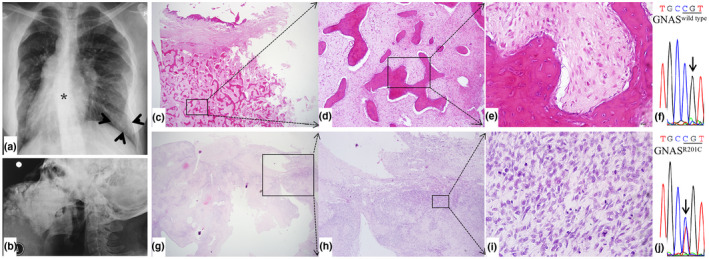
Radiologic‐pathological images and *GNAS* mutation analysis of patient #3. (a) X‐ray chest film showed a radiopaque mass in the rib (arrow heads) and distortion of the vertebra (asterisk). (b) The later left view of the radiologic image shows a giant mass of the mandible. (c–e) Histopathologic images of the benign tissue showed FD features composed of fibrous tissue and irregular bone (c: 4X view, d: 10X view, e: 40X view). (f) *GNAS* mutational analysis showed wild‐type genotype for benign tissues from patient #3. (g–i) Histopathological view of the osteosarcoma region: cellular fibrous stroma and evidence of mineral bone (g: 4X view, h: 10X view, i: 40X view). (j) An R201C mutation of *GNAS* was demonstrated for her malignant tissues

Patient #3 was a 31‐year‐old female, who complained of a progressive increase in mandible swelling for more than 18 years. Except for the mandible lesion, other bone abnormalities were also present, including abnormal ribs (Figure 3a) and both lower limbs, with a history of broken legs. Furthermore, she had precocious puberty and pigmentation, which were signs of MAS. Six months before her referral to our hospital, the mass grew rapidly, with severe pain, breathing problems and numbness of the lower lip. A blood test revealed an elevated ALP level of 1323 U/L. Chest X‐ray revealed curved spines and radiopaque segmental bulging of multiple ribs (Figure 3a). CT examination revealed a huge bone mass of bilateral mandible bodies, with mixed radiopaque‐radiolucent density and resorption of the tooth root. An inferior border of the mandibles was observed (Figure 3b). Resection of the bilateral mandible mass was then carried out, and pathological examination revealed both benign FD lesions (Figure 3c–e) and osteosarcoma (Figure 3g–i).

### 3.2 *GNAS* mutation was detected in both FD and malignant tissues

*GNAS* mutation analysis was performed in both the benign and malignant tissues of each patient. For patient #1 and patient #2, *GNAS* mutation was detected at exon 8, demonstrating c.601 C>T, resulting in the substitution of Arg of 201 to Cys (R201C), a previously reported hotpot activating missense mutation of *GNAS*, in both FD tissue (Figures 1g and 2g) and malignant tissue (Figures 1k and 2k). For patient #3, no mutation was revealed in the benign tissue (Figure 3f), whereas an R201C mutation was found in the malignant tissue (Figure 3j).

### 3.3 WES revealed multiple SNVs and CNAs

**FIGURE 4:**
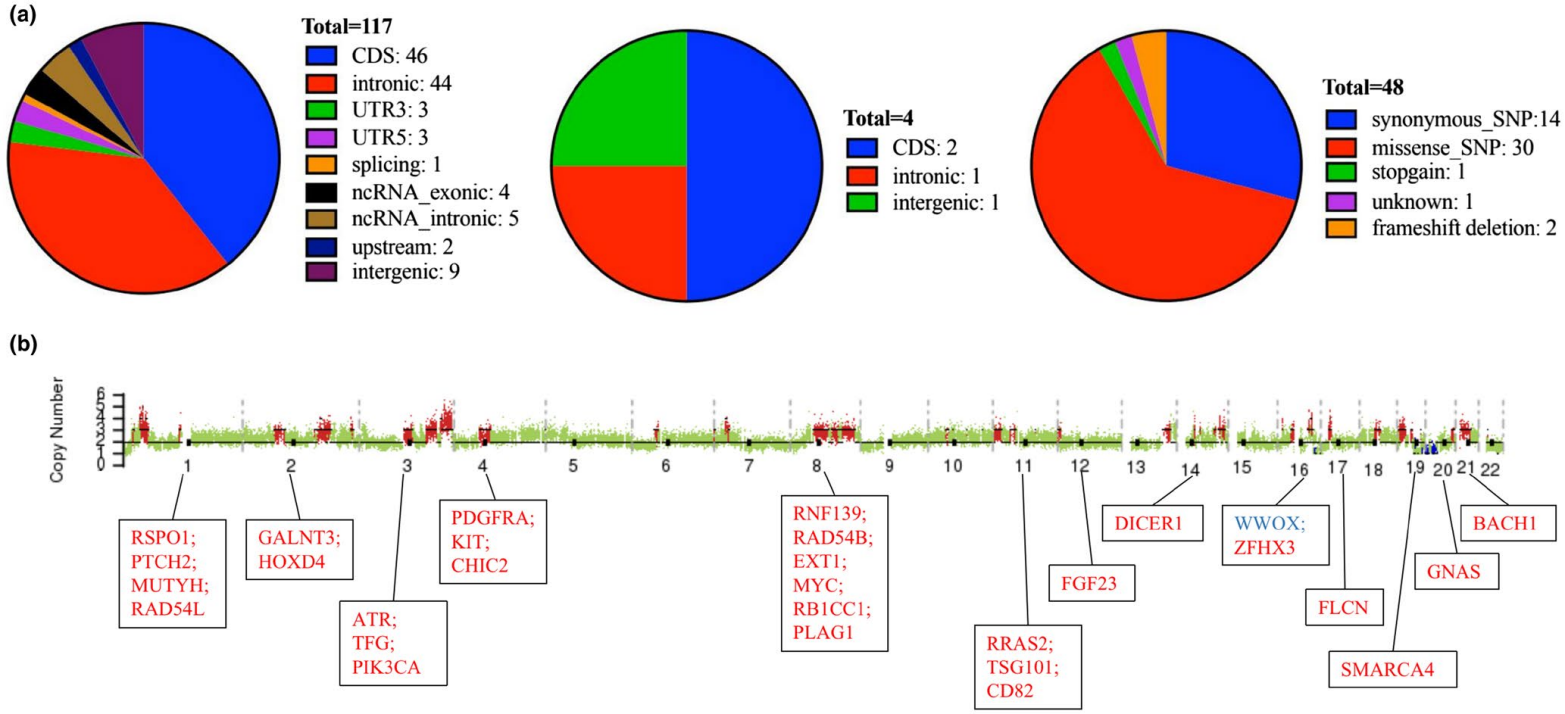
Whole‐exome sequencing information. (a) Summary of somatic SNVs and INDELs specific to malignant tissue. Left: somatic SNV distribution among different regions of the genome. Middle: INDEL distribution among different regions of the genome. Right: SNVs and INDELs in the coding regions. (b) Somatic CNAs specific to the malignant tissue. Amplification of chromosomes 1, 2, 3, 4, 6, 7, 8, 10, 11, 12, 13, 14, 16, 17, 18, 19, 20, 21, 22 and loss of chromosomes 16, 19, 20. Various proto‐oncogenes and suppressor genes related to tumours in the OMIM database were identified and were indicated in rectangles below each chromosome

By comparison with benign FD tissues, we obtained genomic abnormalities specific to malignant tissues, including SNVs and CNAs, using WES. In total, 117 somatic SNVs (Figure 4a, left) and 4 INDELs (Figure 4a, middle) were identified, including 46 SNVs and 2 INDELs in the coding regions (CDSs; Figure 4a, right). By further analysis of genetic aberrations in the CDSs, 30 missense mutations, 2 frameshift deletions and 1 stop‐gain SNV were revealed (Table 2), among which three driver genes were found, including *TP53* (frameshift deletion, NM_000546.6, c.582del), *ROS1* (missense, NM_002944.3, c. T4025>C) and *CDH8* (missense, NM_001170629.2, c.C2586>G). Analysis of somatic CNAs (Figure 4b) revealed multiple chromosomal abnormalities, except for chromosomes 5, 9 and 15. In total, there were 134 gain counts with a size of 413 Mb and 11 loss counts with a size of 20 Mb. In these CNVs, 29 enes were found to have a relationship with tumours in the OMIM database: *RSPO1*, *PTCH1*, *MUTYH* and *RAD54L* on chromosome 1; *GALNT3* and *HOXD4* on chromosome 2; *ATR*, *TFG* and *PIK3CA* on chromosome 3; *PDGFRA*, *KIT* and *CHIC2* on chromosome 4; *RNF139*, *RAD54B*, *EXT1*, *MYC*, *RB1CC1* and *PLAG1* on chromosome 8; *RRAS2*, *TSG101* and *CD82* on chromosome 11; *FGF23* on chromosome 12; *DICER1* on chromosome 14; *WWOX* and *ZFHX3* on chromosome 16; *FLCN* on chromosome 17; *SMARCA4* on chromosome 19; *GNAS* on chromosome 20; and *BACH1* on chromosome 22. Except for *WWOX*, which showed loss, all other 28 genes above were amplified. The details of the somatic CNAs were summarized in Table 3.

**TABLE 2:** The details of somatic nonsynonymous SNVs and INDELs

CytoBand	Position	Ref	Alt	GeneName	Exonic Func	AAChange	dbSNP/COSMIC ID	SIFT	Polyphen 2_HVAR	Polyphen 2_HDIV	Mutation Taster
1p36.12	21016692	C	T	*KIF17*	Missense	NM_001122819.3:c.G1370>A p.(Arg457Gln)	rs186246358	0.019,D	0.032,B	0.144,B	1.000,N
2q31.1	170493312	G	C	*PPIG*	Missense	NM_004792.3:c.G1544>C p.(Arg515Thr)	COSM334392	0.001,D	0.533,P	0.948,P	0.993,D
2q23.1	149539242	C	T	*EPC2*	Stopgain	NM_015630.4:c.C1750>T p.(Gln584Ter)	—	—	—	—	1,A
3p21.31	47043946	A	T	*NBEAL2*	Missense	NM_015175.3:c.A5237>T p.(Asp1746Val)	—	0.002,D	0.999,D	1.0,D	1,D
5q31.2	139060331	C	T	*CXXC5*	Missense	NM_016463.9.:c.C223>T p.(Arg75Cys)	COSM292855	0.001,D	0.642,P	0.999,D	1.000,D
5q31.3	140735915	C	T	*PCDHGA4*	Missense	NM_018917.4:c.C1241>T p.(Thr414Ile)	—	.	0.081,B	0.019,B	1,N
5q35.3	176813546	G	A	*SLC34A1*	Missense	NM_001167579.2:c.G511>A p.(Val171Ile)	rs570463028	0.085,T	0.881,P	0.995,D	1.000,D
6p12.1	54095537	T	A	*MLIP*	Missense	NM_001281747.2:c.T2744>A p.(Val915Asp)	—	0.003,D	0.653,P	0.911,P	1,D
6q22.1	117677908	A	G	*ROS1* ^a^	Missense	NM_002944.3:c.T4025>C p.(Ile1342Thr)	—	0.004,D	0.11,B	0.319,B	0.540,N
7p21.3	8790825	C	T	*NXPH1*	Missense	NM_152745.3:c.C242>T p.(Pro81Leu)	—	0.125,T	0.037,B	0.029,B	1,D
7p15.3	23293048	G	A	*GPNMB*	Missense	NM_001005340.2:c.G193>A p.(Gly65Arg)	—	0.011,D	0.956,D	0.998,D	1,D
7q36.1	150325784	C	T	*GIMAP6*	Missense	NM_001244072.2:c.G112>A p.(Val38Ile)	rs561321166	—	—	—	1,N
8q24.3	145698002	G	A	*KIFC2*	Missense	NM_145754.5:c.G1774>A p.(Ala592Thr)	—	0.405,T	0.017,B	0.11,B	1,N
8q12.3	63902756	CT	C	*NKAIN3*	Frameshift deletion	NM_173688.2:c.563del p.(Leu188fs)	—	—	—	—	—
9q21.11	70176851	A	G	*FOXD4L5*	Missense	NM_001126334.1:c.T1133>C p.(Leu378Pro)	rs3000494; COSM4592895, COSM4592896	1.0,T	0.0,B	0.0,B	0.940,D
10q11.23	49984948	C	A	*WDFY4*	Missense	NM_020945.2:c.C3017>A p.(Thr1006Asn)	—	0.0,D	0.997,D	1.0,D	1.000,D
11q13.3	70333254	G	T	*SHANK2*	Missense	NM_133266.5:c.C1380>A p.(Ser460Arg)	—	0.007,D	0.999,D	1.0,D	1.000,D
12p13.33	1017657	G	A	*WNK1*	Missense	NM_001184985.2:c.G7628>A p.(Arg2543Lys)	—	0.044,D	0.99,D	0.998,D	1.000,D
14q11.2	21876615	G	C	*CHD8* ^a^	Missense	NM_001170629.2:c.C2586>G p.(Phe862Leu)	—	0.001,D	0.883,P	0.924,P	1,D
16p13.3	2570530	G	T	*AMDHD2*	Missense	NM_001145815.2:c.G71>T p.(Gly24Val)	—	0.213,T	0.773,P	0.965,D	0.997,N
16q22.1	67861244	C	T	*TSNAXIP1*	Missense	NM_001288990.3:c.C1757>T p.(Ala586Val)	—	0.42,T	0.124,B	0.279,B	1.000,N
16p11.2	30709563	C	A	*LOC730183*	Missense	NM_001256932.2:c.G67>T p.(Ala23Ser)	—	—	—	—	—
16q12.1	48177945	G	T	*ABCC12*	Missense	NM_033226.3:c.C151>A p.(Leu51Ile)	—	0.009,D	0.945,D	0.988,D	0.723,D
17p11.2	20370767	G	C	*LGALS9B*	Missense	NM_001042685.3:c.C17>G p.(Ser6Cys)	rs4985834, COSM4590741	0.086,T	0.001,B	0.0,B	1,P
17p13.1	7578266	TA	T	*TP53* ^a^	Frameshift deletion	NM_000546.6:c.582del p.(Leu194fs)	COSM308341, COSM308343, COSM308344, COSM308342, COSM308345	—	—	—	—
18q12.2	33828939	G	A	*MOCOS*	Missense	NM_017947.4:c.G2015>A p.(Arg672His)	rs753694626	0.367,T	0.106,B	0.219,B	1.000,N
18q21.1	46447770	C	G	*SMAD7*	Missense	NM_001190821.2:c.G1250>C p.(Trp417Ser)	—	0.0,D	0.998,D	0.999,D	1,D
19p13.3	3961100	G	A	*DAPK3*	Missense	NM_001348.3:c.C689>T p.(Ser230Leu)	rs866486559	0.044,D	0.246,B	0.758,P	1.000,D
19p13.2	10114755	G	C	*COL5A3*	Missense	NM_015719.4:c.C661>G p.(Leu221Val)	—	0.401,T	0.236,B	0.767,P	0.925,N
19q13.11	35434615	C	T	*ZNF30*	Missense	NM_001099437.2:c.C748>T p.(Arg250Trp)	rs373065289	0.08,T	0.015,B	0.069,B	1,N
19q13.43	58863024	G	A	*A1BG*	Missense	NM_130786.4:c.C643>T p.(His215Tyr)	—	0.437,T	0.015,B	0.001,B	1,N
21q21.3	30464016	T	A	*MAP3K7CL*	Missense	NM_001286623.2:c.T161>A p.(Val54Asp)	—	0.008,D	0.144,B	0.902,P	1,N
Xp11.23	46502698	G	A	*SLC9A7*	Missense	NM_001257291.2:c.C1589>T p.(Thr530Ile)	rs940691390	0.003,D	0.943,D	0.992,D	1,D

^a^ Indicate driver mutations.

**TABLE 3:** The details of somatic CNAs

Chr	Start	End	CNV type	GeneName related with OMIM	OMIM
1	36480000	41090000	Gain	*RSPO1*	Palmoplantar hyperkeratosis and true hermaphroditism | Palmoplantar hyperkeratosis with squamous cell carcinoma of skin and sex reversal
1	44090000	46370000	Gain	*PTCH2*	Basal cell carcinoma, somatic; Medulloblastoma
1	44090000	46370000	Gain	*MUTYH*	Adenomas, multiple colorectal; Colorectal adenomatous polyposis, autosomal recessive, with pilomatricomas; Gastric cancer, somatic
1	46370000	47520000	Gain	*RAD54L*	Adenocarcinoma, colonic, somatic (3); Lymphoma, non‐Hodgkin, somatic; {Breast cancer, invasive ductal}
2	163700000	170040000	Gain	*GALNT3*	Tumoral calcinosis, hyperphosphatemic, familial
2	176790000	178310000	Gain	*HOXD4*	{Leukemia, acute lymphoblastic, susceptibility to} (3)
3	140780000	158020000	Gain	*ATR*	Cutaneous telangiectasia and cancer syndrome, familial; GAPO syndrome; Seckel syndrome 1; {Hemangioma, capillary infantile, susceptibility to}
3	93590000	112000000	Gain	*TFG*	Chondrosarcoma, extraskeletal myxoid; Hereditary motor and sensory neuropathy, proximal type
3	178740000	182690000	Gain	*PIK3CA*	Breast cancer, somatic; CLOVE syndrome, somatic; Colorectal cancer, somatic; Cowden syndrome 5; Gastric cancer, somatic; Hepatocellular carcinoma, somatic; Keratosis, seborrheic, somatic; Megalencephaly‐capillary malformation‐polymicrogyria syndrome, somatic; Megalencephaly‐polymicrogyria‐polydactyly‐hydrocephalus syndrome, somatic; Nevus, epidermal, somatic; Nonsmall cell lung cancer, somatic; Ovarian cancer, somatic
4	52930000	75860000	Gain	*PDGFRA*	Gastrointestinal stromal tumor, somatic; Hypereosinophilic syndrome, idiopathic, resistant to imatinib
4	52930000	75860000	Gain	*KIT*	Gastrointestinal stromal tumor, familial; Germ cell tumors; Leukemia, acute myeloid; Mast cell disease; Piebaldism
4	52930000	75860000	Gain	*CHIC2*	{Leukemia, acute myeloid}
8	107740000	134250000	Gain	*RNF139*	Renal cell carcinoma
8	89090000	99230000	Gain	*RAD54B*	Colon adenocarcinoma (3) | Lymphoma, non‐Hodgkin (3)
8	107740000	134250000	Gain	*EXT1*	Chondrosarcoma; Exostoses, multiple, type 1
8	107740000	134250000	Gain	*MYC*	Burkitt lymphoma; Myoclonus, familial cortical
8	49100000	80540000	Gain	*RB1CC1*	Breast cancer, somatic
8	49100000	80540000	Gain	*PLAG1*	Adenomas, salivary gland pleomorphic
11	2920000	18790000	Gain	*RRAS2*	Ovarian carcinoma (3)
11	2920000	18790000	Gain	*TSG101*	Breast cancer, somatic
11	35640000	46010000	Gain	*CD82*	{Prostate cancer, susceptibility to}
12	3640000	4860000	Gain	*FGF23*	Hypophosphatemic rickets, autosomal dominant; Osteomalacia, tumor‐induced (1); Tumoral calcinosis, hyperphosphatemic, familial
14	94780000	99960000	Gain	*DICER1*	Goiter, multinodular 1, with or without Sertoli‐Leydig cell tumors; Pleuropulmonary blastoma
16	77750000	80720000	Loss	*WWOX*	Esophageal squamous cell carcinoma
16	72110000	73170000	Gain	*ZFHX3*	{Prostate cancer, susceptibility to}
17	16390000	18200000	Gain	*FLCN*	Birt‐Hogg‐Dube syndrome; Colorectal cancer, somatic; Pneumothorax, primary spontaneous; Renal carcinoma, chromophobe, somatic
19	5610000	11730000	Gain	*SMARCA4*	Mental retardation, autosomal dominant 16; Rhabdoid tumor predisposition syndrome 2
20	55740000	60080000	Gain	*GNAS*	ACTH‐independent macronodular adrenal hyperplasia; Acromegaly; McCune‐Albright syndrome; Osseous heteroplasia, progressive; Prolonged bleeding time, brachydactyly and mental retardation (3) | Prolonged bleeding time, brachydactyly, and mental retardation (3); Pseudohypoparathyroidism Ia; Pseudohypoparathyroidism Ib; Pseudohypoparathyroidism Ic; Pseudopseudohypoparathyroidism
21	28200000	31730000	Gain	*BACH1*	Breast cancer, early‐onset; Fanconi anemia, complementation group J

Note

GenBank and NCBI reference sequence: *RSPO1*, AK098225.1/NG_012239.2; *PTCH2*, AF091501.1/NG_013369.1; *MUTYH*, U63329.1/NG_008189.1; *RAD54L*, X97795.1/NG_012144.1; *GALNT3*, NG_012069.1; *HOXD4*, NG_012080.1; *ATR*, U76308.1/NG_008951.1; *TFG*, BC009241.2/NG_027821.2; *PIK3CA*, NG_012113.2; *PDGFRA*, D50017.2/NG_009250.1; *KIT*, S67773.1/NG_007456.1; *CHIC2*, AF159423.1/NG_028924.1; *RNF139*, AF064801.1/NG_012158.1; *RAD54B*, AF112481.1/NG_012878.2; *EXT1*, S79639.1/NG_007455.2; *MYC*, NG_007161.2; *RB1CC1*, AB059622.1/NG_015833.2; *PLAG1*, U65002.1/NG_023310.1; *RRAS2*, M31468.1/NG_017058.1; *TSG101*, U82130.1/NG_012138.2; *CD82*, U20770.1/NG_023234.1; *FGF23*, AF263537.1/NG_007087.1; *DICER1*, AB028449.1/NG_016311.1; *WWOX*, AF187015.1/NG_011698.1; *ZFHX3*, D10250.1/NG_013211.2; *FLCN*, AF517523.1/NG_008001.2; *SMARCA4*, D26156.1/NG_011556.3; *GNAS*, AH002748.2/NG_016194.2; *BACH1*, AF026200.1/NG_029658.2.

### 3.4 IHC analysis showed positive staining of p53 in malignant tissues arising from FD

**FIGURE 5:**
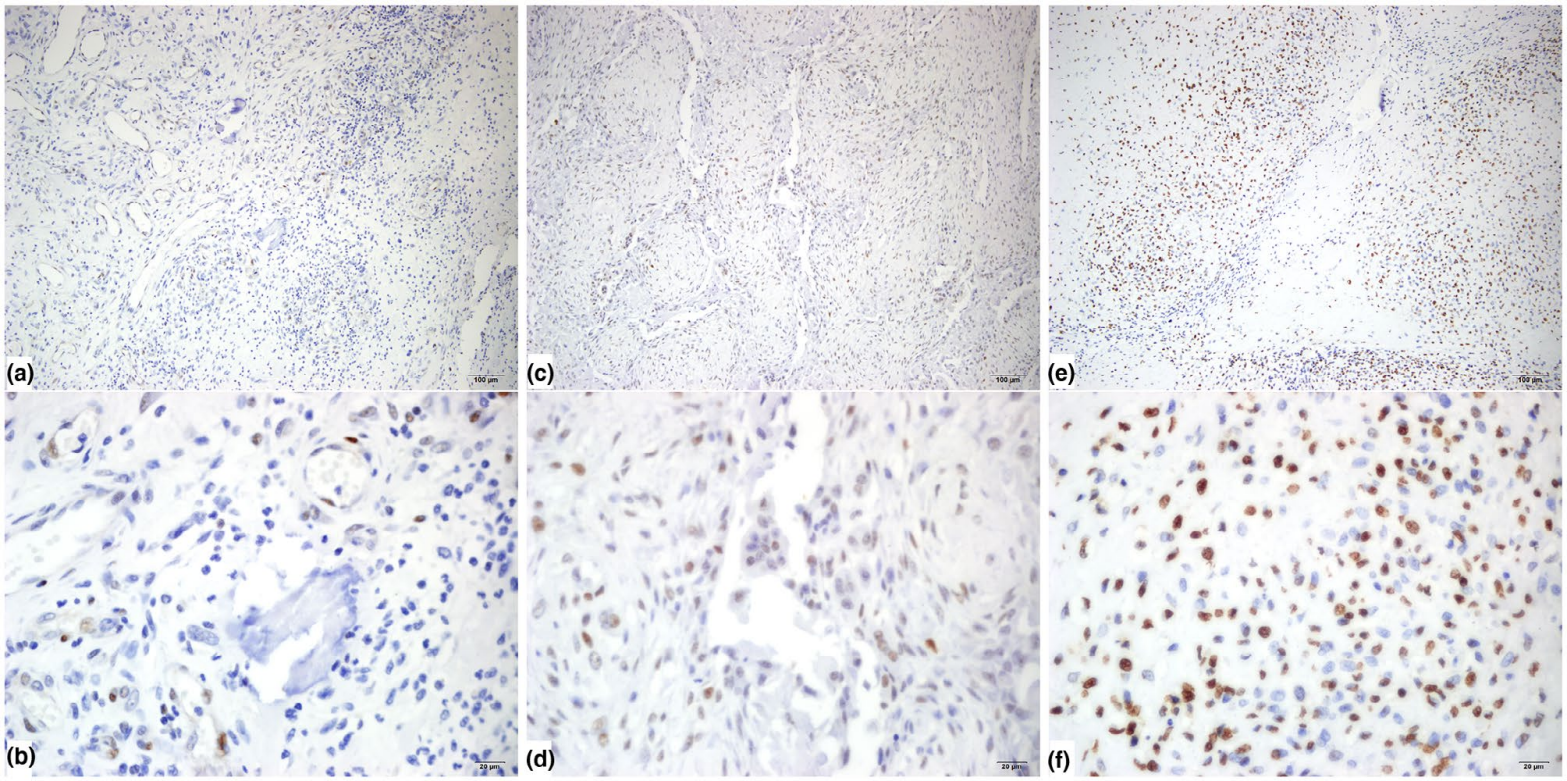
IHC analysis revealed positive expression of p53 in the malignant tissues of all three patients. (a,b) Representative views of patient #1. (a) 10X view, (b), 40X view. (c,d) Representative views of patient #2. (c), 10X view, (d), 40X view. (e,f) Representative views of patient #3. (e) 10X view, (f) 40X view. p53 staining was observed in the nuclei

Alterations in the *TP53* gene in sarcomas often lead to stabilization of the p53 protein and make it visible on IHC, whereas the wild‐type protein has a short half‐life (Yamamoto & Iwakuma, 2018). To test whether there were also *TP53* aberrations in the other two patients without qualified DNA for WES, IHC was performed, which revealed a positive expression of p53 in the malignant tissues of all the patients (Figure 5).

## 4 DISCUSSION

Three well‐documented cases of osteosarcoma arising in FD of the jaws were reported, extending the knowledge of this rare occurrence. More importantly, WES analysis was performed, which revealed significant SNA and CNA involvement on the basis of *GNAS* activating mutations in sarcomas, adding novel and valuable insight into genome alterations underlying FD malignancy.

Although no precise estimation of the frequency of malignant transformation in FD is currently available, it is considered extremely rare. Several groups have made independent estimations based on large populations with FD. In 1964, Schwartz reported a ratio of 0.4%, with 6 cases of sarcoma in a total of 1517 cases of FD (including 1480 cases of monostotic FD and 37 cases of polyostotic FD) and a higher ratio (4%) in a population of 100 patients with MAS (Schwartz & Alpert, 1964). In 1994, a review of Mayo Clinic data revealed 28 cases of malignancy out of 1122 total cases of FD, with a prevalence of ~2% (Ruggieri et al., 1994). More recently, in 2012, three cases of malignancy out of 266 cases in a large Chinese population with craniomaxillofacial FD were identified, revealing a prevalence of 1.1% (Cheng et al., 2012). Similarly, in the present study, we found 3 cases of sarcoma out of 253 cases of FD of the jaws. Therefore, the development of sarcoma from FD is rare. The first well‐documented case was reported in 1945 by Coley and Stewart (1945). A recent review regarding malignant transformation in FD specifically in the craniofacial bones identified only 48 cases in the literature (Li et al., 2020), calling for a larger sample to obtain a better and comprehensive understanding of this entity. In this context, reporting these three new cases in the present study is helpful.

We carefully reviewed the detailed clinicopathological information of these three patients. Moreover, some of the most concerning topics related to this occurrence were reviewed from the literature, including risk factors for malignant transformation, malignant tendency in different sexes and types of FD (monostotic FD, polyostotic FD, MAS) and onset age of sarcoma transformation.

Generally, secondary sarcomas affect male and female patients with FD equally (Li et al., 2020; Ruggieri et al., 1994; Yabut et al., 1988). Similar to the skeletal distribution of FD, craniofacial bones and femur were the most frequently affected sites when sarcomas occurred (Ruggieri et al., 1994; Schwartz & Alpert, 1964). Regarding the onset age of sarcoma occurrence in FD, a wide range of ages were identified, mostly beyond the third decade. The onset ages of all three patients in the present study were all over 30 years old. Interestingly, de novo osteosarcoma from normal bones occurred in jaw bones two decades later than in long bones (Bertin et al., 2020). Whether this rule of primary osteosarcoma is applied to sarcomas secondary to FD is still unknown. We combined and analysed data from two large populations of this specific entity in the literature (Ruggieri et al., 1994; Schwartz & Alpert, 1964) and found that the onset ages for sarcoma were 34.96 ± 16.80 years and 40.22 ± 14.75 years for craniofacial bones and other bones, respectively. There was a site‐related variation in this entity as well; however, statistical analysis (Student's *t* test) showed no significant difference (*p* value: 0.2188).

Regarding potential risk factors leading to malignant degeneration, radiotherapy was the first one suggested in the literature. It was claimed for two reasons. First, radiation itself had carcinogenic effects (Yannopoulos et al., 1964), which can induce primary bone sarcomas (Schwartz & Alpert, 1964). Second, it was originally observed that sarcoma developed in FD exclusively with previous radiation (Schwartz & Alpert, 1964) and specifically in the field of prior irradiation (Ruggieri et al., 1994), which raised the possibility that radiation might play a significant role in sarcoma transformation. However, more cases without prior radiation were reported later (Cheng et al., 2012; Li et al., 2020; Ruggieri et al., 1994; Schwartz & Alpert, 1964) and finding that no major differences in onset age were found between patients with and without radiotherapy (Ruggieri et al., 1994; Schwartz & Alpert, 1964), its role in malignant transformation became weak and controversial. Here, none of the three patients received radiation, again suggesting that there are additional unknown factors contributing to sarcoma development. It has been observed that polyostotic FD and MAS have more malignant potential than monostotic FD (Li et al., 2020; Schwartz & Alpert, 1964). In addition, in a study by Schwartz, the rate of metastasis was reported to be greater in patients with polyostotic FD than in those with monostotic FD (Schwartz & Alpert, 1964). Taken together, polyostotic FD seems to be a high‐risk factor for sarcoma progression. Several case reports have also demonstrated that excess growth hormone (GH; Collins et al., 2012) is associated with malignant transformation and may be a potential driver. Interestingly, patient #1 in the present study showed rapid growth after correction of her hypothyroidism, providing evidence that thyroid‐related hormones may also contribute to malignant transformation.

Based on the above discussion, it is clear that every FD has potential for malignant transformation, regardless of the sex and age of the patient or the location and type of FD. Therefore, all FD patients should be carefully followed up periodically. Specifically, some kinds of FD (polyostotic FD and MAS, especially those with abnormal hormones) have more potential for malignant transformation and need more attention. The role of radiation in malignant transformation in FD is still controversial, and radiotherapy treatment for FD should be avoided.

Very little has been reported in the literature about the genomic and molecular mechanisms underlying sarcoma transformation in FD, with only 7 papers published so far (Hagelstein‐Rotman et al., 2020; Hatano et al., 2014; Jhala et al., 2003; Kanazawa et al., 2009; Sugiura et al., 2018; Yap et al., 2020; Zreik et al., 2017), which are summarized in Table 4. It has been well demonstrated that FD was caused by *GNAS* activating mutations, which can constitutively activate cAMP signalling and result in the abnormal proliferation and osteoblast differentiation of bone marrow stromal cells, the two major features of this disorder. Whether these mutations in FD are retained in malignant tissues has drawn much attention. In the literature, *GNAS* mutation analysis of malignant tissues has been performed in only 7 patients, and 5 of them showed mutations, with 3 R201C (p.Arg201Cys; Kanazawa et al., 2009; Yap et al., 2020; Zreik et al., 2017) and 2 R201H (p.Arg201His; Hatano et al., 2014; Sugiura et al., 2018). In the present study, we detected R201C *GNAS* mutations in all three patients. Collectively, *GNAS* mutations were retained in most of the sarcomas (8/10), with more R201C (75%) than R201H (25%) mutations. Whether a sarcoma in patients with FD comes from pre‐existing FD or de novo has been controversial. Since the evaluation by Schwartz, which revealed that sarcomas always developed in bones affected by FD (Schwartz & Alpert, 1964), the view of sarcomas originating from pre‐existing FD has become less controversial and has become more widely accepted. However, direct evidence of sarcoma development in FD has been lacking for a long time. The finding that *GNAS* mutations exist only in FD other than primary osteosarcoma (Salinas‐Souza et al., 2015; Tabareau‐Delalande et al., 2013) provides an opportunity to study the origin of sarcomas in FD patients. The combined data from the literature and our study now allow us to make more confident conclusions that FD has malignant potential.

**TABLE 4:** Published genetic mechanisms underlying FD malignancy in literature

Country	Sample size	Onset age of malignancy (years)	Sex	Site	Type of FD	Malignant pathology	Genetic mechanisms for sarcoma transformation		
							Detection of *GNAS* mutation in the malignant tissues	Other molecular mechanisms	Methods for other molecular analysis
US (Jhala et al., 2003)	1	44	F	Right elbow	Concommitant MAS and Mazabraud's syndrome	OS	Not included	Chr5 and Chr7 trisomies, multiple chromosomal abnormalities	G‐banded karyotype on short‐term primary cells, FISH on paraffin‐embedded tissues, CGH of DNA from paraffin‐embedded tissues
Japan (Kanazawa et al., 2009)	1	38	F	Mandible	MAS	OS	R201C mutation	Expression of c‐fos and PTH/PTHrP; excess serum GH and IGF‐1	Immunohistochemistry on paraffin‐embedded tissues; laboratory examinations
Japan (Hatano et al., 2014)	1	72	M	Femur	PFD	OS	R201H mutation	44,X,‑Y, add(4)(p11), add(5)(p15), der(11)add(11)(p15)t(1;11)(q21;q23),add(12)(q11), ‑13, der(22)t(12;22)(q11;p12)	G‐banded karyotype
US (Zreik et al., 2017)	1	45	M	Femur	PFD	NS	R201C mutation	Aberrant expression of multiple karatins	Immunohistochemistry
Japan (Sugiura et al., 2018)	1	33	M	Hip joint	MFD	OS	R201H mutation	Positive staining for p53 and MDM2; MIB‐1 index: 15%; negative for CDK4; no *MDM2* amplification	Immunohistochemistry and FISH on paraffin‐embedded tissues
Netherlands (Hagelstein‐Rotman et al., 2020)	7	NS	NS	NS	4 MFD, 3 NS	OS	2 out of 7 underwent *GNAS* mutation detection, with one negative and one inconclusive	Not included	not included
Australia (Yap et al., 2020)	1	21	M	Maxilla	MFD	OS	R201C mutation	*TP53* mutation (Asp281Asn); positive staining for p53; Ki‐67 proliferation index: 25%; no *MDM2*/*CDK4* amplification	Next‐generation sequencing; immunohistochemistry; FISH

Abbreviations: F, female; M, male; MAS, MuCune‐Albright syndrome; MFD, monostotic fibrous dysplasia; NS, not specified; OS, osteosarcoma; PFD, polyostotic fibrous dysplasia; R201C, p.Arg201Cys; R201H, p.Arg201His.

In addition to the *GNAS* mutation analysis above, other molecular mechanisms underlying sarcoma development in FD, including aberrant *TP53* (Sugiura et al., 2018; Yap et al., 2020), multiple chromosomal abnormalities (Hatano et al., 2014; Jhala et al., 2003), increased cell proliferation (Sugiura et al., 2018; Yap et al., 2020), MDM2 (Sugiura et al., 2018), c‐fos (Kanazawa et al., 2009), PTH/PTHrP (Kanazawa et al., 2009) and karatins (Zreik et al., 2017), have been reported in case reports using traditional chromosome abnormality methods and IHC, which can reveal only limited information. Next‐generation sequencing has illuminated rich and unappreciated genomic alterations for various diseases; however, only one study using WES analysis for malignant transformation in FD was found in the literature (Yap et al., 2020) when we prepared for the present study. Through WES analysis, we found several valuable genomic aberrations for FD malignancy. First, three‐driver genes were found in the malignant tissues associated with FD: *ROS1*, *CHD8* and *TP53*. Among them, *TP53* is the only one that has been reported in the literature for this occurrence. However, different from the reported *TP53* point mutation (Asp281Asn; Yap et al., 2020), it was an INDEL in the present study. Functionally, *TP53* is a tumour suppressor gene that is essential for regulating cell division and preventing tumour formation. According to a recent study (Sayles et al., 2019), *TP53* gain‐of‐function alterations can be detected in 74% of osteosarcoma cases. We also confirmed the abnormality of *TP53* by IHC in the other two patients here. Taking the data from the literature and our present study, it is obvious that similar to primary osteosarcoma, *TP53* abnormalities may be a significant event in osteosarcoma secondary to FD.

Regarding *TP53* aberration in the malignant transformation of FD, it is notable that its coexistence with *GNAS* activating mutations has been reported in other malignant tumours, including colorectal adenocarcinoma, oesophageal squamous cell carcinoma, pancreatic adenocarcinoma and non‐small‐cell lung cancer (Table 5, data from TCGA database). Furthermore, in a recent study (Patra et al., 2018), malignant transformation (pancreatic ductal adenocarcinomas, PDAs) of a benign pancreatic disorder, intraductal papillary mucinous neoplasm (IPMN), induced by *GNAS* mutation, with the addition of *TP53* loss was reported, supporting the significant role of *TP53* in the progression of benign disease with pre‐existing *GNAS* mutation to a malignant state. Whether this coexistence of *TP53* and *GNAS* variations makes the osteosarcoma different from the counterpart without *GNAS* mutation is unclear. Pathologically, no significant difference could be found, with all three of our cases being the conventional OS. However, based on the surprising finding by Patra et al that mutant *GNAS* was still required for the maintenance and growth of tumours after malignant transformation of IPMN, it is probably true that GNAS mutation also retained an important role after FD malignancy. Therefore, it might be feasible to treat secondary malignancies from FD and other diseases bearing this mutation by gene targeting therapy in the future. Indeed, molecular therapy targeting *GNAS* activating mutations has been demonstrated to be effective for FD in vitro (Piersanti et al., 2010).

**TABLE 5:** Coexistence of *GNAS* activating mutation and *TP53* aberration (TCGA database)

Study ID	Sample ID	Patient ID	Disease	*GNAS*	*TP53*
coadread_tcga_pan_can_atlas_2018	TCGA‐AG‐3732‐01	TCGA‐AG‐3732	Colorectal adenocarcinoma	R201L	K164Sfs*5
coadread_tcga_pan_can_atlas_2018	TCGA‐WS‐AB45‐01	TCGA‐WS‐AB45	Colorectal adenocarcinoma	R201H, P329Qfs*361	R175C
esca_tcga_pan_can_atlas_2018	TCGA‐JY‐A6FG‐01	TCGA‐JY‐A6FG	Esophageal squamous cell carcinoma	R201H	R273H
luad_tcga_pan_can_atlas_2018	TCGA‐55‐8089‐01	TCGA‐55‐8089	Non‐small cell lung cancer	R201C	R249S
luad_tcga_pan_can_atlas_2018	TCGA‐78‐7540‐01	TCGA‐78‐7540	Non‐small cell lung cancer	R201H	R273C
luad_tcga_pan_can_atlas_2018	TCGA‐95‐7567‐01	TCGA‐95‐7567	Non‐small cell lung cancer	R201H	Y205H
paad_tcga_pan_can_atlas_2018	TCGA‐HV‐AA8V‐01	TCGA‐HV‐AA8V	Pancreatic adenocarcinoma	R201C	P151R
stad_tcga_pan_can_atlas_2018	TCGA‐BR‐7707‐01	TCGA‐BR‐7707	Esophagogastric adenocarcinoma	R201C	N131del

Note

GenBank and NCBI reference sequence: *GNAS*, AH002748.2/NG_016194.2/NM_000516.7; *TP53*, AF307851.1/NG_017013.2/NM_000546.6.

Somatic CNAs are another important aspect underlying the genetic mechanism of primary osteosarcoma. It has even been hypothesized that CNAs rather than point mutations may be the dominant oncogenic mechanism for osteosarcoma progression and maintenance (Sayles et al., 2019). Furthermore, Sayles et al. (2019) identified some somatic CNAs most likely to be of direct clinical relevance, targeting these somatic CNAs could lead to a significant decrease in tumour burden. Somatic CNAs across cases of osteosarcoma are highly complex and heterogeneous; thus, their analysis in each patient is of great importance. In the literature, only one paper has demonstrated the gain of chromosomal regions in FD malignancy using comparative genomic hybridization (CGH) analysis (Jhala et al., 2003). In the present study, through WES, more detailed information about somatic CNVs in a secondary osteosarcoma of FD was revealed. Various oncogenes and tumour suppressors, including *KIT*, *PDGFRA*, *MYC*, *EXT1*, *PIK3A* and *WWOX*, are located in these somatic CNAs. Based on Sayles's study, targeting oncogenes within these CNAs may provide hope for the treatment of patients with these specific genomic abnormalities.

## CONFLICT OF INTERESTS

The authors declare that they have no conflict of interest.

## AUTHOR CONTRIBUTIONS

Study design: RRS, TJL. Study procedures and data collection: RRS, XFL, MM, YRF. Data analysis and interpretation: RRS, XFL, JYZ, FC, TJL. Manuscript preparation: RRS, FC, TJL.

## ETHICAL COMPLIANCE

This study was approved by the Institutional Review Board of Peking University and conducted in accordance with the Declaration of Helsinki.

## Data Availability

The data that support the findings of this study are available from the corresponding author upon reasonable request.
